# Mechanosensitive components of integrin adhesions: Role of vinculin

**DOI:** 10.1016/j.yexcr.2015.11.017

**Published:** 2016-04-10

**Authors:** Paul Atherton, Ben Stutchbury, Devina Jethwa, Christoph Ballestrem

**Affiliations:** Wellcome Trust Centre for Cell-Matrix Research, University of Manchester, Manchester M13 9PT, UK

**Keywords:** Vinculin, Actin, Focal adhesion, Mechanotransduction, Force

## Abstract

External forces play a key role in shaping development and normal physiology. Aberrant responses to forces, or changes in the nature of such forces, are implicated in a variety of diseases. Cells contain several types of adhesions, linking them to their external environment. It is through these adhesions that forces are both sensed (from the outside inwards) and applied (from inside to out). Furthermore, several adhesion-based proteins are sensitive to changes in intracellular forces, utilising them for activation and regulation. Here, we outline how vinculin, a key component of integrin-mediated adhesions linking the actin cytoskeleton to the extracellular matrix (ECM), is regulated by force and acts as force transducing protein. We discuss the role of vinculin *in vivo* and its place in health and disease; summarise the proposed mechanisms by which vinculin is recruited to and activated at integrin-ECM adhesions; and discuss recent findings that place vinculin as the major force sensing and transmitting component of cell–matrix adhesion complexes. Finally, we discuss the role of vinculin in regulating the cellular responses to both the physical properties of the external environment and to externally applied physical stimuli.

## 1 Introduction

The human body can be thought of as a collection of chemical signalling events, intertwining to regulate physiological processes. However, the large collection of tissues and organs comprising the human anatomy has a second key regulator: exposure to the physical environment of the tissue and to ever-changing mechanical stimuli. Human anatomy consists of a myriad of tissues, ranging from the very soft gyri and sulci of the brain, to the very hard rigid trabeculae of bones. In addition to this heterogeneity, tissues continuously experience environmental forces, generated by blood pressure and movement. Understanding how the cells that constitute these tissues sense and respond to this multitude of mechanical stimuli is becoming ever important, and is known to regulate development and tissue homeostasis. It is now apparent that the response to force occurs at a protein level and the underlying molecular mechanisms are under intense investigation.

The 117 kDa focal adhesion protein vinculin (from the Latin *vinculum* meaning “bond”, “link” or “tie”) was discovered in 1979 as a protein localizing at the distal ends of microfilament bundles at the cell membrane [25]. Since its initial discovery, vinculin has become one of the best-characterised proteins of the focal adhesion (FA) where it has emerged as one of the main components of the mechanosensory machinery. Recent advances in microscopy have allowed us to gain a deeper insight into the precise location of vinculin within a FA. Elegant super-resolution microscopy experiments have placed vinculin within a ‘force-transduction layer’ where it links actin filaments to the extracellular matrix (ECM), through talin and integrin [10], [35]. This imaging work supports functional molecular studies that show separate roles for the head domain of vinculin in regulating integrins (through its association with talin) and of the tail in regulating the link to the actomyosin machinery [30].

In this review we focus on the role of vinculin *in vivo*; its force-dependent recruitment and activation within cells; how vinculin acts to transmit forces from inside the cell to the extracellular matrix; and the regulation of the cellular response to mechanical stimuli by vinculin. In addition to its function at FAs, recent work has highlighted a role for vinculin at cell–cell junctions (reviewed by DeMali et al. [20]).

## 2 Role of vinculin *in vivo*

Genetic ablation of vinculin in mice is embryonic lethal, with defects seen by day E8 and termination by E10. Specific abnormalities are seen in the development of the nervous system, most likely due to defects in cell migration, and also in the developing heart [55]. Interestingly, vinculin appears to have a key function in the heart (a tissue under considerable forces), which fits with its role as a mechanosensitive protein. Vinculin was observed to localize to the intercalated discs joining cardiomyocytes [40] and knock-down of vinculin in primary cardiac myocytes disrupted cell shape and the alignment and assembly of myofibrils [48]. Furthermore, a missense mutation within the vinculin gene that reduced the levels of the protein within the intercalated discs was identified in a patient with hypertrophic cardiomyopathy [53].

Much of the work to elucidate the role of vinculin in cardiac tissue has been done using animal models (reviewed in detail by Zemljic-Harpf et al. [58]), where authors have shown that cardiac-specific knockout of vinculin leads to either sudden death or the development of dilated cardiomyopathy [59]. Additionally, artificially inducing stress to the hearts of heterozygous vinculin knockout mice led to the development of cardiomyopathy [60]. More recently, vinculin has been identified as a regulator of cardiac function during aging, with overexpression of vinculin being protective against stress and increasing the lifespan of *D. melanogaster* by >150% [36].

Whilst these studies clearly demonstrate that vinculin is involved in the adaptation of tissues to forces, the ability of vinculin to regulate the actin cytoskeleton also appears to be important for normal homeostasis of bone tissue. Bone resorption is driven by osteoclasts at actin-rich structures known as the sealing zone. Osteoclast-specific knockout of vinculin in mice led to smaller sealing zones and increased bone mass, with the cellular phenotype rescued by expression of wild-type vinculin, but not by expression of actin binding deficient mutants [24].

Taken together, the *in vivo* data shows a clear function of vinculin in both regulating adaptations to forces and in regulating the actin cytoskeleton. These roles are reflected at the molecular level, where vinculin is regulated by intracellular forces and is also involved in force transduction, and at the cellular level, where vinculin regulates cellular responses to mechanical stimuli.

## 3 Mechanisms of recruitment and activation of vinculin

In cells plated on stiff 2D substrates, integrin-dependent cell–matrix interactions form at the leading edge as focal complexes (FX) and mature into FAs under actomyosin-mediated tension. Both tension independent FX, as well as tension dependent FAs, contain vinculin [57] and several models of how vinculin becomes recruited to these sites have been proposed, including force-dependent and force-independent mechanisms. Most of these models are based on the initial biochemical characterisation of vinculin by Johnson and Craig [34] which revealed that vinculin is formed of three functional groups: the head, neck and tail domains. Bakolitsa et al. [5] determined that the full-length, 1066 amino acid structure is formed of 5 domains. *In vitro*, domains 1–3 of the head form a ‘pincer’, holding domain 5 (the tail) tightly bound. This conformation is not able to bind to talin suggesting it is an autoinhibitory, inactive conformation [17]. Point mutations to disrupt this auto-inhibition lead to increased binding of talin (*via* the head domain) and actin at the tail domain. These biochemistry results suggest that when vinculin is activated *in vivo*, the autoinhibitory bond is broken leading to the unmasking of a number of binding sites for other FA proteins [9]. The precise mechanism(s) underpinning how vinculin in cells is recruited to adhesion sites and how the autoinhibitory bond is broken remain open to discussion and will be outlined below.

### 3.1 Recruitment by talin alone

A prerequisite for FA formation and for vinculin recruitment to FAs is the presence of talin. Talin has 11 vinculin binding sites (VBS), the majority of which are thought to be cryptic, requiring specific unmasking events [19]. Talin is clearly the major binding partner for vinculin in FAs; while talin forms the essential structural link between integrin and the intracellular actomyosin machinery, vinculin reinforces this link [1], [30]. Integrin-talin engagement at nascent adhesions induces an enrichment of PIP_2_ in the inner membrane leaflet [41], and PIP_2_ binding to talin is one proposed mechanism for talin’s activation; indeed, sequestering PIP_2_ disrupts FAs [43]. Actomyosin-mediated forces across talin are proposed to unmask VBSs [8] and early studies suggested that, once activated, talin alone is sufficient to activate vinculin [31]. However, this model has been questioned, as the autoinhibitory interaction between the vinculin head and tail is extremely strong (Kd<1 nM) and a single ligand is considered insufficient to break this bond for full activation of vinculin. Furthermore, *in vitro*, the talin rod only binds to vinculin constructs lacking the auto-inhibitory bond [17]. These results suggest there is a requirement for another vinculin binding partner for the vinculin–talin interaction to occur, and the currently preferred model includes a combination of signals for vinculin activation.

### 3.2 Recruitment by talin and PIP_2_

**Fig. 1:**
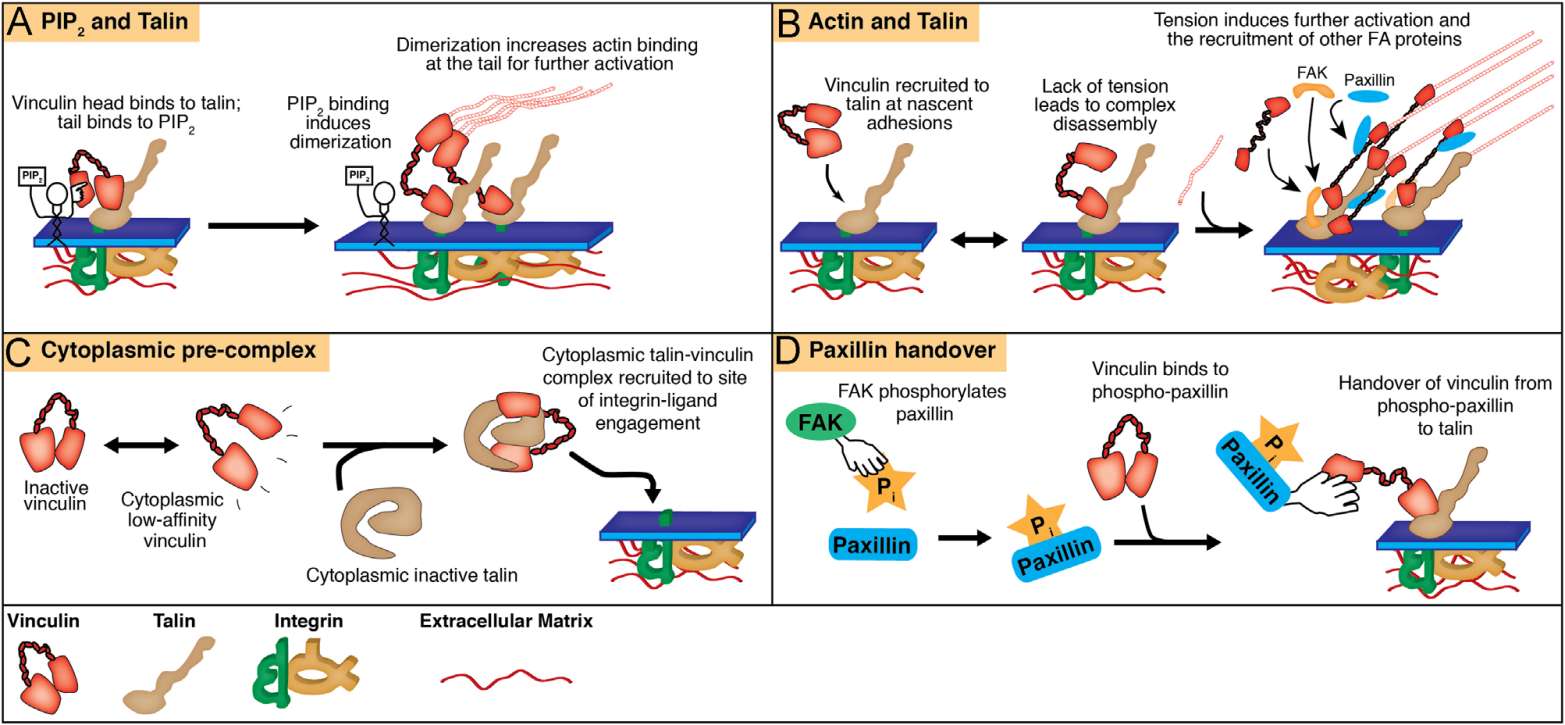
Models of vinculin recruitment and activation. Four potential mechanisms have been proposed to contribute to vinculin recruitment to integrin-ECM adhesion sites and activation. While these have been separated here, it is likely that a combination of these mechanisms is used in the cell. A. Vinculin binds to integrin-bound talin *via* the head domain. PIP_2_, which is enriched at these sites, binds to the vinculin tail leading to dimerization and increasing actin binding. B. Vinculin is recruited to talin bound to the cytoplasmic tail of integrin, inducing partial activation. Actin binding at the tail, providing actomyosin-based tension, is required for further activation of vinculin; without actin binding, the two proteins dissociate and the nascent adhesion does not mature. C. Vinculin undergoes rapid conformational changes in its tertiary structure, switching between an inactive and a low-affinity state. The low affinity state is able to bind to cytoplasmic talin (itself in either an inactive state, or also in a ‘low-affinity’ state (not shown)) to form a cytoplasmic ‘pre-complex’, which is then recruited to sites of integrin-ligand engagement. D. Paxillin is phosphorylated by FAK at nascent adhesions. Vinculin binds to phosphorylated paxillin, which then ‘hands over’ vinculin to integrin-bound talin.

The vinculin tail domain contains a binding site for PIP_2_ (phosphatidylinositol 4,5-bisphosphate) [6] and early studies suggested that this interaction, together with talin binding at the vinculin head, could activate vinculin [26]. This model was reinforced by the finding that PIP_2_ binding at the vinculin tail could induce a conformational change in vinculin, possibly forming the first step towards full activation [6], [26]. However, more recent studies have questioned the validity of this model acting *in vivo*; vinculin constructs containing mutations disrupting lipid binding (Vinculin-LD) still localise to FAs, but their expression reduces cell motility [15]. These findings led to the hypothesis that uncoupling of the vinculin tail from the ‘pincer-like’ head domains might occur through PIP_2_ competing with actin binding at the vinculin tail [11], [49]. However, it was recently shown that PIP_2_ binding induces structural rearrangements of vinculin leading to dimerization, thus amplifying binding to actin [14], [15]. Therefore, despite initially being thought to pre-activate vinculin, PIP_2_ is now believed to be a critical factor for modulating its actin binding potential (see Fig. 1a). Indeed, interfering with PIP_2_ binding results in defects in actin stress fibre organisation, cell spreading and migration [15].

### 3.3 Recruitment by talin and activation by actin

PIP_2_ is not the only molecule proposed to have an auxiliary role in the talin-mediated activation of vinculin. The combination of talin and actin was proposed to be able to break the auto-inhibitory head-tail bond of vinculin [5]. Such a model was supported by the findings that neither a talin peptide that mimiced an activated vinculin binding site on the talin rod (VBS3) nor actin alone, but rather the presence of the two together were able to bind vinculin and change its conformation to an activated state [13]. Therefore, vinculin at nascent adhesions may undergo rapid on/off cycles of talin binding, requiring actin and subsequent force application for further activation (see Fig. 1b); PIP_2_ binding may promote this actin–vinculin association (as discussed above), leading to the complete activation of vinculin. More recently, the phosphorylation of vinculin has been proposed to play a role in modifying the binding of vinculin to actin after initial talin binding, and this may be an additional regulatory step [2].

### 3.4 Formation of a cytoplasmic vinculin–talin complex and recruitment to adhesions

Despite the reservations of the early hypothesis of activation by talin alone, a more recent finding has demonstrated that talin and vinculin can form a complex in the cytoplasm, which is then recruited to integrin at nascent adhesions [3]. This discovery is perhaps not surprising, given that proteins undergo rapid conformational fluctuations in their tertiary structures, a process known as “breathing” [39]. In this model, either vinculin or talin (which also has an auto-inhibitory bond [8]) would undergo a rapid conformational change leading to the relaxation of auto-inhibition, thus allowing either talin or vinculin binding respectively (see Fig. 1c).

### 3.5 Recruitment of vinculin by phosphorylated paxillin

An alternative mechanism for vinculin recruitment to adhesions has been put forward by Pasapera et al. [44], who revealed that myosin II-dependent phosphorylation of paxillin by FAK was involved in vinculin binding. In this model, phosphorylated paxillin would hand vinculin over to the adjacent talin molecule (see Fig. 1d). Once ‘transferred’ from phospho-paxillin to talin, actin and PIP_2_ may further induce vinculin activation and FA stabilisation. This potential role of paxillin has recently been further supported using super-resolution microscopy, where inactive vinculin was recruited to FAs *via* direct interactions with phosphorylated paxillin [10].

## 4 Force dependent regulation of vinculin at FAs

Once in the FA, vinculin remains sensitive to forces. Photokinetic experiments using fluorescence recovery after photobleaching (FRAP) show that vinculin does not simply bind to and remain in the FA; rather individual FA proteins exist in a continual state of flux, undergoing cycles of activation and dissociation from the complex. Constitutively active forms of vinculin (either with mutations blocking head-tail interactions (vinT12), or truncation removing the auto-inhibitory binding tail (vin880 and vin258)) have a reduced rate of turnover compared to the wild-type protein [18], [30], implying a tighter association within the FA. The expression of these constitutively active forms of vinculin leads to larger FAs [30], mimicking a high-force environment [7]. The opposite effect is seen upon the addition of blebbistatin, which leads to a loss of intracellular tension. In these conditions, the rate of dissociation of vinculin from the FA is more rapid, indicating a weaker association with the FA [54].

In addition to influencing the turnover rate of vinculin, intracellular tension is also required to maintain vinculin in FAs. A FRET-based vinculin activation reporter localises predominantly to FAs, suggesting that actomyosin-mediated tension stabilises vinculin in the activated state [18]. Treatment of cells with either blebbistatin or the Rho-associated protein kinase (ROCK) inhibitor Y-27632 releases intracellular tension due to inhibition of myosin II-mediated actomyosin contractility. Under these conditions, vinculin is lost from adhesions within as little as 30 minutes of drug treatment. Constitutively active vinculin mutants are impervious to the effects of tension ablation and remain at FAs after drug treatment, but this is dependent on their ability to bind talin [9], highlighting the importance of force for the association of these two key FA structural components. In addition, FRAP experiments conducted during treatment with Y-27632 (*i.e.* as FAs disassemble) show almost no recovery of fluorescence after bleaching; indicating that tension also plays a role in the recruitment of vinculin to FAs [9]. Analysing the co-localisation of several other FA proteins with various constitutively active vinculin mutants, in the absence of intracellular tension, showed that vinculin acts as a master orchestrator of the FA, regulating the recruitment and release of several FA proteins in a force-dependent manner [9].

Together, these studies show that intracellular tension contributes not only to vinculin’s activation and recruitment to the FA, but also to its role in regulating the adhesion complex as a whole.

## 5 Vinculin transmits forces from inside the cell to the ECM

Focal adhesions transmit intracellular, myosin-generated forces from the cytoskeleton to the ECM, generating traction forces that pull the cell body forward during cell migration [51]. Vinculin's position in the FA, with its head bound to talin and tail bound to actin, means it is ideally placed for the transmission of intracellular forces to the ECM. Indeed, vinculin-deficient fibroblasts have a 4-fold reduction in tractional forces, demonstrating that vinculin has a key role in this process [21].

Dumbauld and colleagues [23] demonstrated that the vinculin–actin interaction is required for force transmission: inducing myosin contractility increased cell adhesion strength in a vinculin-dependent manner and vinculin-deficient cells have reduced adhesion strength and are insensitive to inhibitors of myosin contractility, such as blebbistatin. This suggests that vinculin is directly affected by actomyosin contractility and is responsible for transmitting strain from actin through the FA to the ECM.

These studies, implicating vinculin in the transmission of force from actin through to the ECM, are supported by the use of a vinculin-FRET probe that was used to determine the amount of tension across the protein This showed vinculin to be under ~2.5 pN of tension in stable FAs [27]. This vinculin tension sensor was then used to demonstrate that the tension across vinculin was reduced upon the laser ablation of actin stress fibres [12]. Moreover, a study using Vin^−/−^ MEFs expressing various vinculin mutants showed that the vinculin-head, most likely through its interaction with talin, was responsible for enhancing adhesion strength; whereas the actin-binding tail was required for the generation of vinculin-dependent traction forces [22].

Myosin generated tension, coupled with front-end polymerisation of actin filaments, leads to the process of actin retrograde flow. It is the transmission of this motion through focal adhesions that leads to cellular traction forces [51]. Actin-binding FA proteins such as vinculin and talin also undergo retrograde flow [29]; a tail-less form of vinculin, in contrast to a full-length constitutively active form, did not undergo retrograde flow, demonstrating the requirement of vinculin’s actin binding site in this process [30]. Follow up work has demonstrated that vinculin slows the rate of actin retrograde flow at focal adhesions, leading to the generation of high traction forces; a vinculin point mutation (I997A) reducing binding to F-actin blocked the retardation of retrograde flow [51]. Expression of vinculin containing a second recently identified mutation in the actin binding site [33] reduced traction force generation, indicating that force from the retrograde flow of actin is transmitted through the actin binding site of vinculin to the ECM.

Together, these findings highlight the importance of both the vinculin–talin interaction and the vinculin–actin interaction for the transmission of intracellular forces to the ECM. It appears that talin-bound vinculin can act as a molecular clutch, engaging F-actin undergoing myosin mediated retrograde flow and transmitting this motion as traction force to the underlying ECM [51].

### 5.1 Responses to the mechanical ECM environment

The transmission of actomyosin-generated tension from the inside of the cell to the outside through FAs exerts force upon the extracellular environment, generating an opposing, resistance force; the vinculin–actin link is proposed to act to ‘sense’ the resistance from this opposing force, thus regulating intracellular signalling events. By using high-resolution traction force microscopy, Plotnikov et al. [45] measured with high accuracy the spatial and temporal exertion of traction forces upon a substrate by cells at FAs. Surprisingly, such force applications were found to exist in two states, either stable or fluctuating. The fluctuating forces were linked to repeated ‘tugging’ of the ECM, and the authors demonstrated that such tugging was involved in the sensing of ECM rigidity and regulated durotaxis, the movement of cells from lower to higher rigidity substrates. Consistent with its role in linking the ECM to actin, vinculin was found to be integral to this process, acting *via* a phospho-paxillin/FAK pathway to enable ECM rigidity sensing.

Yamashita et al. [56] have also proposed a mechanism by which vinculin senses ECM rigidity. Here, the authors showed that cells on more rigid substrates formed more FAs, which are also larger, than on softer substrates, and that this was blocked by depletion of vinexin-α. Vinexin binds at a proline-rich region within the neck domain of vinculin. Mutation of this binding site, or depletion of vinculin or vinexin-α, blocked the stiffness-dependent increases in cell velocity seen in wild-type cells. Taken together, these two studies outline a pathway whereby vinculin, linked to vinexin, senses ECM rigidity and signals through paxillin and FAK to regulate cell motility. The precise role that vinexin plays in this mechanosensing is unclear; the binding of vinexin to vinculin may alter the conformation of vinculin to permit mechanosensing, or the signalling responses may be driven specifically by vinexin, which contains three SH3 domains and is capable of regulating the cytoskeleton and cell adhesion [37].

How might these molecular mechanisms of ECM-rigidity sensing affect cell behaviour *in vivo*? The differentiation of human mesenchymal stem cells (MSCs) to a muscle lineage is dependent on high ECM-rigidity. Consistent with the molecular studies described above, knock-down of vinculin was found to reduce the expression of myoD, a transcription factor driving the commitment of MSCs to a skeletal muscle lineage, and their subsequent differentiation to muscle cells [28]. Rigidity sensing is also important in cancer metastasis: the dissemination of cells from one tissue type to another is likely to be associated with alterations to the cancer cells’ ECM environment and rigidity. Furthermore, tumours within a tissue often have a different rigidity to the surrounding healthy tissue, which is likely to affect both cancer cells and non-malignant stromal cells [32]. Again, vinculin appears to have a role in this: Rubashkin et al. [46] demonstrated that increased ECM stiffness stabilized the assembly of a vinculin–talin–actin scaffolding complex, regulating PI3K-mediated Akt signalling within FAs, thus promoting tumour progression.

It should be noted that rigidity is only one physical property of the ECM, and other attributes such as ECM porosity and the orientation of ECM-proteins can also play a role in influencing cellular behaviour [42]. Whether vinculin at cell-ECM adhesions also plays a role in sensing these physical elements of the ECM remains to be determined. In addition, *how* these physical properties are altered in disease and aging and how this affects cellular behaviour, are two closely linked topics that demand further research.

### 5.2 Responses to externally applied forces

In addition to responding to the physical properties of the ECM, cells also respond to rapid and transiently applied forces, such as those generated *in vivo* by the cardiovascular system. Stretching a cell’s substrate is a transient force, and vinculin has been shown to be responsible for regulating Rac activation and subsequent cell polarisation in response to such stimulation [9]. Experiments using fibronectin-coated magnetic beads to apply a controlled force to adhesions have shown that such forces lead to cell stiffening in a vinculin-dependent manner, requiring the actin-binding site of vinculin [52]. Cell stiffening is regulated by Rho-family GTPases and has been observed to change with ECM stiffness in a FA-signalling dependent manner [4].

### 5.3 Vinculin directs feedback mechanisms regulating intracellular signalling

**Fig. 2:**
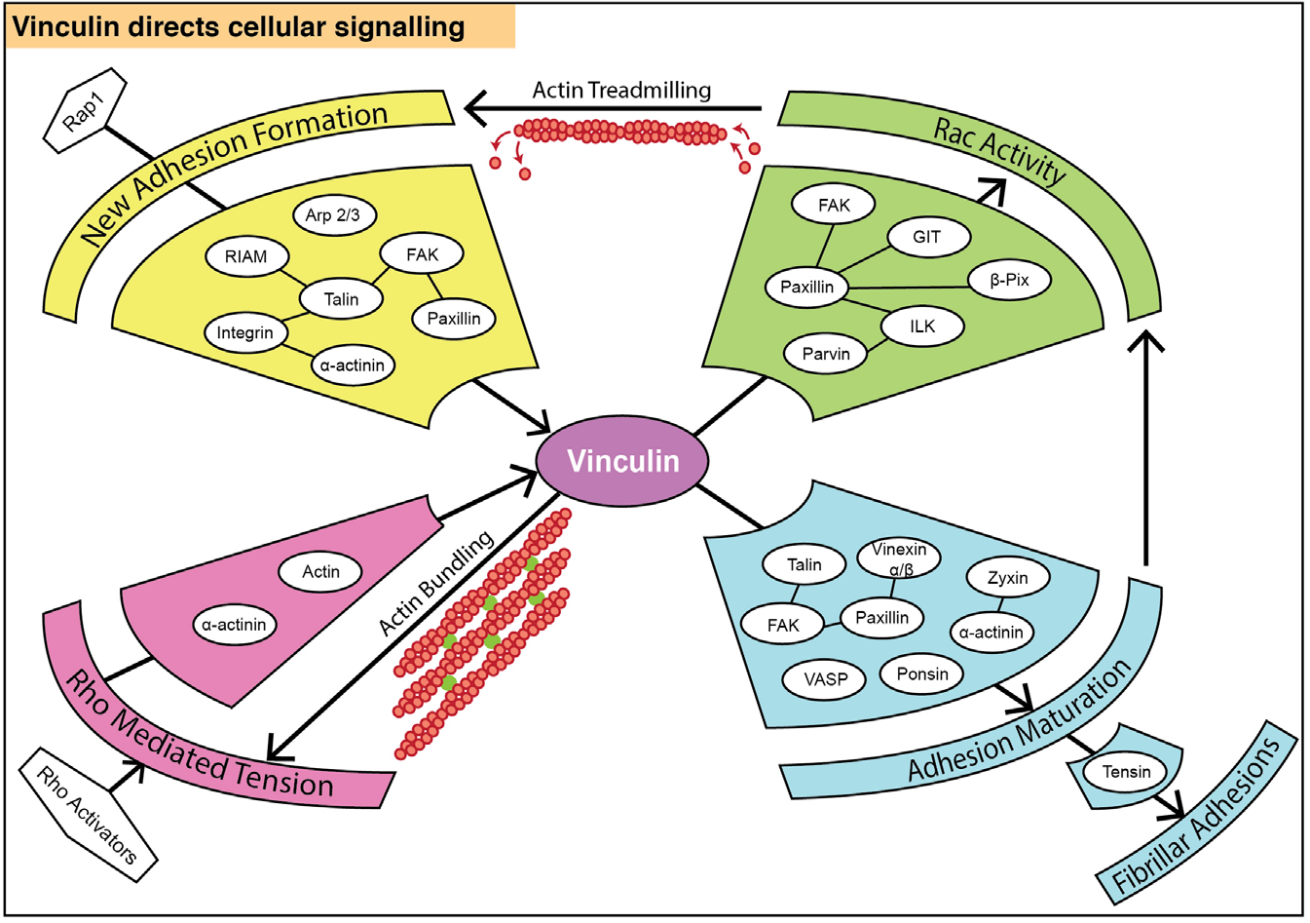
Vinculin directs intracellular signalling and responds to intracellular tension. Proteins that bind, either directly or indirectly, to vinculin are indicated in white circles. Vinculin binds to several proteins capable of regulating the activity of Rac (for example, paxillin and FAK), which promotes the formation of new adhesions at the leading edge. The formation of new adhesions recruits proteins such as talin and paxillin, which are implicated in the recruitment and activation of vinculin as discussed earlier. Vinculin is also capable of bundling actin filaments and is itself regulated by intracellular tension.

It is apparent that vinculin has two key roles in mechanosensing: (1) transmission of actomyosin-generated intracellular tension through the FA to the ECM; (2) ‘sensing’ the resistance to this force, or other externally applied forces, by regulating intracellular signalling events in response. Indeed, vinculin lies at the centre of an intricate signalling network (Fig. 2). It is able to bind to and/or regulate proteins that initiate the formation of new adhesions at the active-Rac-driven leading edge, such as the Arp2/3 complex [16]. It also regulates proteins involved in the generation of intracellular tension *via* Rho activation and the bundling of actin [33], leading to the maturation of focal complexes to FAs or FAs to fibrillar adhesions. Adhesion maturation also leads to the recruitment of vinculin-binding partners (and indeed, further vinculin) capable of driving Rac activity, thus promoting new adhesion formation. In two-dimensional culture, cells lacking vinculin showed faster migratory speeds compared to wild-type cells [33], [47], suggesting that vinculin has a role in modulating GTPase activity to regulate motility. In a three-dimensional environment the effects of vinculin knock-out differ somewhat, with vinculin appearing to regulate directional migration (persistance) rather than speed [50]. These differences between 2-D and 3-D culture highlight our need to improve our understanding of how FA-signalling is modulated by the ECM environment.

## 6 Conclusions

In the past decade the field of cellular mechanobiology has exploded. It is now clear that as well as receiving chemical signals cells are also able to sense and respond to mechanical, force-generated signals from their environment. In this review, we have described how vinculin has rapidly become the central figure in cellular mechanosensing and how it is vital, not only for cellular homeostasis, but also to maintain healthy function at a physiological level.

What may the future hold for this small but powerful protein? Analysing the specific interactions of vinculin will allow us to further our understanding of tissue homeostasis and diseases, particularly in conditions where forces are known to contribute. A central theme throughout this review has been the force-dependent interactions between vinculin, talin and actin, comprising a mechanosensitive module of the FA. It is important to note that vinculin at FAs has an intimate relationship with talin [8]. This was recently shown to be a key regulator of force-dependent development in *Drosophila*, whereby vinculin regulates the orientation of talin relative to integrin, thus regulating force sensing [38]. Further work will allow the specific interactions between these proteins to be dissected, in order to further understand the complex feedback signals involved in cellular mechanosensing. Moreover, the recently identified role of vinculin at mechanosensitive cell–cell junctions [20] highlights an intriguing possibility that vinculin may orchestrate feedback mechanisms between cell–cell junctions and cell–ECM adhesions.
